# A booster with SARS-CoV-2 vaccines: protection against Omicron infection

**DOI:** 10.1038/s41392-022-00973-5

**Published:** 2022-04-05

**Authors:** Yuehong Hu, Qiu Sun

**Affiliations:** 1 State Key Laboratory of Biotherapy, Cancer Center, West China Hospital, Sichuan University and Collaborative Innovation Center for Biotherapy, 610041 Chengdu, China; 2 Department of Pediatric Pulmonology and Immunology, West China Second University Hospital, Sichuan University and NHC Key Laboratory of Chronobiology, 610041 Chengdu, China

**Keywords:** Infection, Immunotherapy

Recently, Wang et al. published a study in *Nature* that examined whether sera from individuals who received two or three doses of the inactivated vaccine could neutralize Omicron.^1^ Broad-spectrum and potent neutralizing antibodies were isolated from three-dose recipients, which could effectively neutralize strain pseudoviruses and authentic viruses of SARS-CoV-2 of concern (VOCs), including Omicron. In addition, the cryo-EM structures of the Omicron spike were elucidated, revealing a new critical immune evasion site and mechanism for the Omicron strain.^2^

Since Omicron reduced the replication fitness of the virus itself for a better immune escape ability in the immune host, Omicron variant escaped almost all clinically approved antibody therapeutics and possessed higher transmission rates among household contacts than Delta, which raised unprecedented concerns of antibody therapies and vaccines. Previous studies have shown that prolonged and repeated antigen stimulation could drive ongoing antibody somatic mutation and antibody composition changes, which were an increased proportion of broad-spectrum antibodies in the B memory cell repertoire, conferring the development of monoclonal antibodies with enhanced neutralizing potency and breadth.^3^ Therefore, it was speculated that the three-dose vaccine may still be effective in protecting against Omicron infection.

**Fig. 1:**
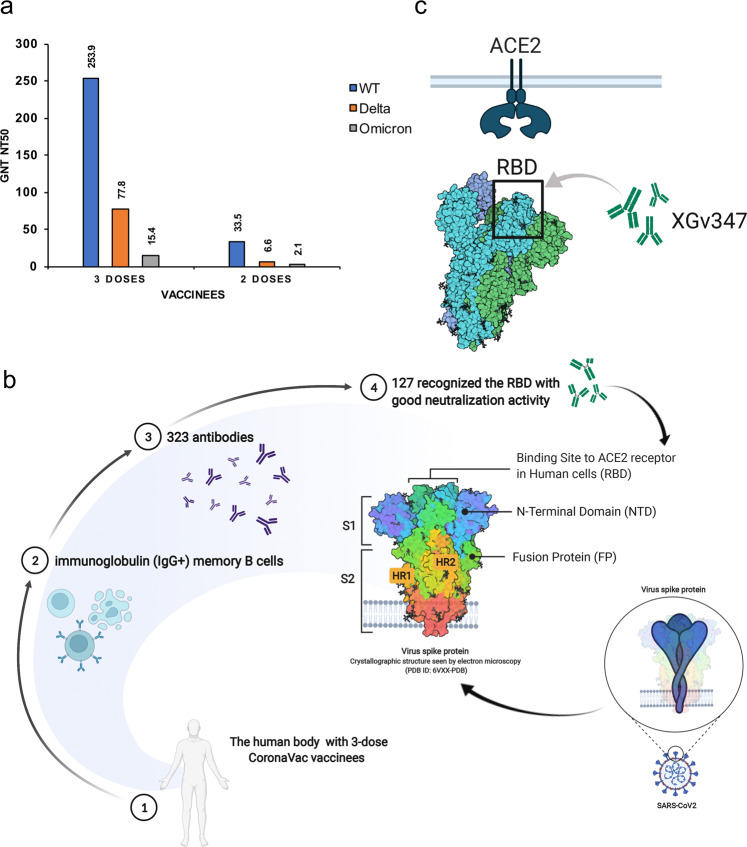
**a** The GMT NT50 against VOCs from vaccinated volunteers with two and three doses. The neutralizations against the WT, Delta, and Omicron variants are shown as blue column, orange column, and gray column, respectively, the data are reproduced from the original article^1^ with permissions. **b** The flow chart of the study.^1^ **c** XGv347 activation mode. The model depicts a possible mechanism that XGv347 bound to an epitope at the tip of the RBD, largely overlapping with the patch targeted by ACE2. This figure was prepared using BioRender (https://biorender.com/)

This study tested serum specimens from vaccinated volunteers for neutralizing antibody titers against the wild-type (WT) virus, Delta, and Omicron variants using live SARS-CoV-2 first. The results showed that among two-dose CoronaVac recipients, the geometric mean half-maximal neutralizing titers (GMT NT50) were only 33 against live WT virus, 6 against Delta, and almost 0 against Omicron. However, it was more concerning that for three doses of CoronaVac recipients, the NT50 against live WT virus increased to 253.9 and against Delta and Omicron variants decreased by 3.3 and 16.5 times to 77.8 and 15.4, respectively (Fig. 1a). These results showed that sera of the three-dose vaccines displayed higher neutralization titers against VOCs than the two-dose vaccines. However, the NT50 against the Omicron variants was low, even for the three-dose vaccines.

Furthermore, 1800 SARS-CoV-2 S-specific memory B cells from peripheral blood mononuclear cells (PBMCs) were sorted from people vaccinated with three-dose CoronaVac. The sequences of the antibody heavy and light chain variable regions were obtained, and 323 antibodies were expressed in vitro (Fig. 1b). They found that more than 90% of the antibodies had an affinity with the original strain S protein at a sub-nM level. 127 RBD-directed antibodies showed good neutralization activity against both authentic and pseudotyped WT SARS-CoV-2, of which 31 RBD antibodies showed broad-spectrum neutralization activity against the original strain and 5 VOCs, including Omicron, and 24 antibodies neutralized Omicron with IC50 values were lower than 0.1 μg/ml. These results suggested that ~7% of broadly neutralizing antibodies were present in the repertoire of specific memory B cells generated by the three-dose vaccine. When the body is exposed to Omicron or new mutant strains in the future, this subset of memory B cells will be specifically activated and differentiated into plasma cells that secrete high-efficiency antibodies, suggesting that clinical protection against severe Omicron infection may still be effective with a three-dose vaccine at the molecular level.

By analyzing complex structures of SARS-CoV-2-neutralizing antibodies and RBD antigens from the PDB database, they classified neutralizing antibodies into classes I–VI according to the different antigen-binding sites on the RBD. It was identified that broad-spectrum potent RBD-neutralizing antibodies mainly belong to classes I, II, and IV. Antibodies XGv347 (class II) and XGv289 (class IV) had neutralizing activity against all VOCs. Both XGv282 (class IV) and XGv265 (class IV) had high neutralizing activity against VOCs except for Omicron. In addition, XGv265 had more significantly reduced neutralizing activity against Omicron compare with XGv282.

Interestingly, the cryo-EM complex structures of the Fab region of the above four antibodies with Omicron S revealed that the Fab of XGv347 bound to Omicron S in three distinct conformational states, whereas the others had only one state. XGv347 bound to an epitope at the tip of the RBD, largely overlapping with the patch targeted by ACE2 (Fig. 1c). Structural comparisons revealed that XGv347 had epitopes similar to A23-58.1 and S2K146, which were broad-spectrum antibodies reported previously.^4^ Furthermore, XGv347 exhibited a good protective effect against Omicron infection, and no obvious lung pathological injury was observed in the mouse model. These results suggested a plausible capability of XGv347 to cross-neutralize Omicron and circulate SARS-CoV-2 variants. Structural comparative analysis revealed two new key antibody escape sites, G446S and N440K, for Omicron. Given the presence of G446S and N440K mutations, the binding and neutralizing activity of antibodies class IV, such as XGv289, XGv282, and XGv265, which are known to highly neutralize most VOCs, might decrease to varying degrees against Omicron.

It is noteworthy that among these four representative antibodies, compared to WT, XGv282 showed a minimal reduction in binding affinity (fivefold) to Omicron RBD. However, the binding affinity of XGv347 to Omicron decreased 40-fold, whereas the neutralizing activity of XGv347 against Omicron was almost unchanged. These results suggested that compared with the binding affinity, the epitope of the antibody binding to the antigen has a greater impact on the neutralizing potency and breadth of an antibody. Therefore, the epitope is a critical factor in the neutralizing antibody selection process.

The same group also resolved the structure of the apo Omicron S trimer and found that the Omicron S trimer was in a highly stable “active” state, maintaining the conformation bound to the receptor ACE2 at all times, and its binding affinity to the receptor was enhanced by approximately threefold.^2^ Some new hydrogen bonds or salt bridges were formed for key amino acid mutations on the RBD.^5^ These changes in the local conformation, charge, and hydrophobic microenvironment underpin the modulation of epitopes so that they were not recognized by most NTD and RBD antibodies. All these results suggested that the stability and attachment of Omicron S were enhanced and that the ability of viral immune escape was promoted. Finally, the authors systematically analyzed the coronavirus, including SARS-CoV, SARS-CoV-2 variants, civet, bat, and pangolin-derived sarbecoviruses, and revealed the intrinsic rules, key amino acid sites, and conformational characteristics of coronavirus binding to human ACE2.

To sum up, this study systematically screened, identified, and classified the SARS-CoV-2-neutralizing antibodies that can broadly neutralize WT and all VOCs. Different types of antibodies can form a cocktail therapy to prevent and treat Omicron/future variants infection. In addition, the results also provide a theoretical basis for the protection of severe infection of the Omicron by the three-dose vaccine and rationalize the use of a booster with SARS-CoV-2 vaccines. These results are cross-validated with the abovementioned structural features, providing a theoretical basis for developing broad-spectrum vaccines and immunotherapy.
